# Determinanten der Gesundheit und medizinischen Versorgung von Asylsuchenden in Deutschland

**DOI:** 10.1007/s00103-023-03762-9

**Published:** 2023-09-14

**Authors:** Amand Führer

**Affiliations:** Institut für Medizinische Epidemiologie, Biometrie und Informatik, Profilzentrum Gesundheitswissenschaften, Martin-Luther-Universität Halle-Wittenberg, Magdeburger Straße 8, 06112 Halle (Saale), Deutschland

**Keywords:** Asyl, Psychische Gesundheit, Zugangsbarrieren, Sammelunterkünfte, Diskriminierung, Asylum, Mental health, Barriers to access healthcare, Refugee shelters, Discrimination

## Abstract

Asylsuchende sind in Deutschland einer Vielzahl gesundheitlicher Belastungen ausgesetzt, während ihr Zugang zu medizinischer Versorgung erschwert ist. Diese Übersichtsarbeit erläutert einige Determinanten, die diese Situation strukturieren, und führt dabei u. a. aus, wie sich das Asylbewerberleistungsgesetz (AsylbLG), die Abrechnung über Behandlungsscheine und die Unterbringung in Sammelunterkünften auf die Gesundheit von Asylsuchenden und ihre Inanspruchnahme medizinischer Versorgung auswirken. Hierbei wird deutlich, dass die Ausgliederung von Asylsuchenden aus dem Sozialsystem der Gesundheit der Betroffenen schadet, teuer ist und zudem ethische und rechtliche Fragen aufwirft. Insbesondere der große Ermessensspielraum der Sozialämter in der Kostenübernahme sowie die unterschiedlichen Modelle zur Abrechnung medizinischer Leistungen sind gerechtigkeitstheoretisch problematisch und führen zu einer Versorgungspluralität, die der rechtlichen und ethischen Begründung entbehrt.

Im zweiten Teil des Artikels wird dargestellt, dass Asylsuchende im Allgemeinen dieselben gesundheitlichen Probleme haben wie gesetzlich Krankenversicherte – mit der Ausnahme von psychischen Erkrankungen, die bei Asylsuchenden deutlich häufiger auftreten, oft jedoch nicht diagnostiziert und häufig nur unzureichend behandelt werden.

Als Schlussfolgerung für die Praxis leitet sich ab, 1) dass Asylsuchende in die gesetzliche Krankenversicherung integriert werden sollten, 2) dass aus sozialmedizinischer Sicht eine Unterbringung in eigenen Wohnungen der Unterbringung in Sammelunterkünften vorzuziehen ist und 3) dass das Gesundheitssystem Anpassungsleistungen erbringen muss, um eine diversitätssensible Versorgung aller Patient/-innen sicherzustellen.

## Abstract (en)

Asylum seekers in Germany are exposed to a variety of health-related stressors, while their access to medical care is impaired. This review explains some of the determinants that structure this situation, for example by elaborating on how the Asylum Seekers’ Benefits Act (ASBA), billing via treatment vouchers, and accommodation in refugee shelters affect asylum seekers’ health and healthcare utilization. Hereby, it becomes clear that the exclusion of asylum seekers from the welfare system is detrimental to their health, is expensive, and raises ethical and legal questions. In particular, the huge discretionary scope of the social welfare offices in the assumption of costs as well as the different models for billing medical services for asylum seekers are potentially unjust and lead to a plurality of care, which lacks legal and ethical justification.

The second part of the article shows that asylum seekers generally suffer from the same health problems as people with statutory health insurance—with the exception of mental illnesses, which are substantially more common among asylum seekers, but are often undiagnosed and frequently inadequately treated.

This leads to three practical conclusions: (1) asylum seekers should be integrated into statutory health insurance, (2) from a public health point of view, accommodation in refugee shelters should be avoided in favor of decentralized accommodation, and (3) to ensure diversity-sensitive care for all patients, the healthcare system must make adjustments to its structures and practices.

## Hintergrund

Deutschland verfügt über das älteste staatliche Krankenversicherungssystem der Welt, dessen Entwicklung maßgeblich durch eine kontinuierliche Ausweitung der eingeschlossenen Bevölkerungsgruppen gekennzeichnet war: Während in der Frühphase die Versicherungspflicht vor allem die Versorgung von Fabrikarbeiter/-innen sicherstellte, weitete sich das durch staatliche Sicherungssysteme aufgespannte Netz in der Folge immer weiter und schloss immer größere Teile der Bevölkerung ein [1].

Dieser Logik der Ausweitung sozialer Sicherungssysteme läuft seit den 1980er-Jahren der zunehmende Ausschluss von Asylsuchenden aus dem deutschen Sozialsystem zuwider. Mit dem sogenannten Asylkompromiss beschloss die Bundesregierung 1992 nicht nur massive Einschränkungen im Asylrecht, sondern gliederte Menschen während des Asylverfahrens und mit Duldung auch aus dem Bezug von Sozialleistungen aus. Anstelle der Sozialgesetzbücher wurde mit dem Asylbewerberleistungsgesetz (AsylbLG) ein neues Gesetz verabschiedet, welches die für Asylsuchende zugänglichen Leistungen regelt, dabei aber nicht die soziale Sicherung, sondern die Migrationskontrolle als vordergründiges Ziel verfolgte [2, 3].

Diese Übersichtsarbeit^1^ hat zum Ziel, diese Sonderstellung anhand der vorhandenen Literatur darzustellen und ihre Implikationen für die medizinische Versorgung zu skizzieren. Dabei werden zunächst wesentliche Einflüsse erläutert, die die Gesundheit von Asylsuchenden^2^ strukturieren, und dann die gesundheitliche Situation dieser Bevölkerungsgruppe dargestellt. Im Fazit werden einige Schlussfolgerungen für die Praxis abgeleitet.

## Determinanten der Gesundheit und medizinischen Versorgung von Asylsuchenden

Die Gesundheit und medizinische Versorgung von Asylsuchenden werden durch verschiedene Einflussfaktoren strukturiert, die sich zum Teil von den Einflussfaktoren unterscheiden, die für die gesetzlich krankenversicherte Bevölkerung prägend sind [6]. Hierbei ist zu unterscheiden zwischen Determinanten, die spezifisch für Asylsuchende sind, und solchen, die sich aus ihrer Migrationsgeschichte ergeben und die sie mit anderen Gruppen von Migrant/-innen teilen. Im Folgenden wird der Fokus auf Erstere gesetzt, während Letztere nur skizziert werden. Eine ausführliche Darstellung der Determinanten der Gesundheit von Migrant/-innen findet sich in dem entsprechenden Beitrag in diesem Heft.

### Statusspezifische Determinanten

Asylsuchende sind in Deutschland einer Reihe rechtlicher Sonderregeln unterworfen, die sich aus ihrem Aufenthaltsstatus ergeben: Ihr Zugang zum Arbeitsmarkt ist eingeschränkt; sie können ihren Wohnort und ihre Wohnung nicht frei wählen; ihr Recht auf Familiennachzug ist aufgehoben; sie unterliegen bestimmten Meldepflichten und sie haben keinen Anspruch auf Sozialleistungen, sondern beziehen reduzierte Leistungen nach dem AsylbLG [7].

Diese rechtliche Sonderstellung wurde in den 1980er- und 1990er-Jahren in mehreren gesetzgeberischen Schritten hergestellt, welche 1993 im AsylbLG vorerst kulminierten [8]. Ziel der damaligen Gesetzgebung war es, Deutschland als Migrationsziel möglichst unattraktiv zu machen und durch eine Absenkung des für Asylsuchende zugänglichen Leistungsniveaus Anreize zur Migration nach Deutschland zu mindern und gleichzeitig Kosten zu sparen [2, 3].^3^ Während alle der oben genannten Aspekte mittelbar gesundheitsrelevant sind, sollen sich die folgenden Ausführungen auf 3 Bereiche konzentrieren, zu denen bereits umfassend Literatur mit Gesundheitsbezug vorliegt: das Asylbewerberleistungsgesetz, die Abrechnung medizinischer Leistungen und die Wohnbedingungen von Asylsuchenden.

### Das Asylbewerberleistungsgesetz

Die im AsylbLG definierten Regeln gelten aktuell für die ersten 18 Monate des Aufenthaltes in Deutschland oder bis eine Aufenthaltserlaubnis erteilt wird. In diesem Zeitraum rechnen behandelnde Ärztinnen und Ärzte mit dem zuständigen Sozialamt (und nicht der Krankenkasse) ab [11, 12].

Der Kanon der durch das Sozialamt zu erstattenden medizinischen Leistungen ergibt sich dabei aus 2 Paragraphen des AsylbLG, den Paragraphen 4 und 6: Im § 4 AsylbLG wird die Kostenübernahme für die Behandlung von akuten und schmerzhaften Erkrankungen, Impfungen, Vorsorgeuntersuchungen und für Behandlungen im Zusammenhang mit Schwangerschaft und Geburt festgelegt, womit eine medizinische Basisversorgung garantiert werden soll [13, 14]. Darüber hinausgehende Leistungen wie die Versorgung nichtprogredienter chronischer Erkrankungen, Hilfs- und Heilmittel, Krankentransport oder Dolmetscher können nach § 6 AsylbLG ebenfalls gewährt werden, sofern sie „im Einzelfall zur Sicherung … der Gesundheit unerläßlich“ sind.

Der an sich juristisch und (medizin‑)ethisch bereits hoch problematische Ansatz, Asylsuchende aus dem Bezug von Sozialleistungen auszugliedern [15, 16], wird dadurch noch problematischer, dass beide für die medizinische Versorgung unmittelbar relevanten Paragraphen durch ein hohes Maß an Ermessensspielraum gekennzeichnet sind: Einerseits ist der Tatbestand der „akuten Erkrankung“ juristisch nicht definiert, sodass im Einzelfall fraglich ist, welche Behandlungsanlässe von § 4 erfasst sind. Andererseits ist auf der Rechtsfolgenseite die Ausgestaltung der „sonstigen Leistungen“ nicht konkretisiert und deren Gewährung zudem in das Entschließungsermessen des Sozialamtes gestellt [17].

Dieses für das deutsche Recht ungewöhnlich große Maß an Ermessen birgt die Gefahr, dass durch Unterschiede in der Ausgestaltung des Ermessens die Rechtssicherheit leidet. Dass unterschiedliche Sozialämter ihr Ermessen unterschiedlich nutzen, führt dazu, dass in vielen Fällen der „Zufall über den Zugang zur Gesundheitsversorgung bestimmt“ [18], was gerechtigkeitstheoretische Fragen aufwirft [19].

Um dem entgegenzuwirken, gilt der Grundsatz, dass die Ausgestaltung von Ermessen an der Absicht des Gesetzgebers orientiert sein soll [17, 20]. Bezogen auf das AsylbLG ist dieser Grundsatz aber in zweifacher Hinsicht problematisch:

Erstens zeichnet sich das AsylbLG im Vergleich zu anderen Gesetzen durch ein besonders hohes Maß an Ermessen und unbestimmten Tatbeständen aus. Dies erklärt sich daraus, dass das Ermessen in diesem Fall nicht primär auf die Sicherstellung von Einzelfallgerechtigkeit abzielt, sondern vielmehr der politischen Kontroverse um das Gesetz Rechnung trägt [17]. Offensichtlich war die Formulierung des Gesetzestextes von der Absicht getragen, politisch nicht konsensfähige Entscheidungen an die Verwaltung und den Einzelfall zu delegieren [21, 22]. Dadurch fehlt dem AsylbLG ein klar formulierter Wille des Gesetzgebers als Richtschnur für die Ausgestaltung des Ermessens und der diskretionären Macht der Verwaltung [22]. Schon aus theoretischen Überlegungen heraus muss daher eine „große Vielfalt der Umsetzungspraxis“ [22] vermutet werden, die sich in empirischen Studien auch bestätigt [18, 23].

Zweitens hat sich durch die jüngere Rechtsentwicklung der juristische Kontext des AsylbLG geändert, wodurch eine Neuinterpretation dessen nötig wird, was als vermutete Absicht des Gesetzgebers gelten soll und daher als Interpretationsleitlinie zur Orientierung des Ermessens herangezogen werden muss [24, 25]: So urteilte das Bundesverfassungsgericht im Jahr 2012, dass das AsylbLG in seiner damaligen Form verfassungswidrig war und die migrationspolitisch motivierte Einschränkung von Leistungen nicht mit der im Grundgesetz garantierten Menschenwürde zu vereinbaren ist [2]. Zudem ergibt sich aus der Ratifizierung der EU-Aufnahmerichtlinie die Notwendigkeit, das deutsche Recht mit dem EU-Recht zu harmonisieren [26]. Da dies bis zum Ablauf der Umsetzungsfrist im Juli 2015 nicht erfolgt ist, sehen richtungsweisende Urteile mehrerer Landessozialgerichte die Notwendigkeit, dass das Ermessen in der Anwendung des AsylbLG nun so genutzt werden muss, dass Verfassungs- und Richtlinienkonformität hergestellt wird [27, 28]. Dies bedeutet in der Praxis, dass der Kanon der „sonstigen Leistungen“ sich regelhaft am Leistungskanon der Gesetzlichen Krankenversicherung (GKV) zu orientieren hat [25].

Im Alltag zeigt sich jedoch, dass diese jüngere Rechtsentwicklung von vielen Sozialämtern nicht umgesetzt wird [29], was zu ausgeprägten regionalen Unterschieden in der Kostenübernahmepraxis und in der Folge zu regionalen Unterschieden im Kanon der vom Kostenträger übernommenen medizinischen Leistungen führt [18].

Neben der Beförderung regionaler Ungleichheit wirkt die mit der Kostenübernahme verbundene Unsicherheit zudem in die Ärzteschaft hinein und befördert dort die Wahrnehmung, dass Asylsuchende eine „andere Kategorie“ von Patienten darstellen, deren Versorgung separaten Prinzipien folgt. Im Ergebnis lässt sich beobachten, dass Ärztinnen und Ärzte ihren Patient/-innen unter Verweis auf das AsylbLG Leistungen verweigern, auf die diese einen Rechtsanspruch haben [23, 30].

### Die Abrechnung medizinischer Leistungen

Neben den regionalen Unterschieden im Umgang mit dem Ermessen im AsylbLG gibt es auch in der Organisation der Abrechnung von Leistungen und – damit verbunden – der Organisation des Zugangs zum Gesundheitssystem verschiedene Ansätze: die Abrechnung über Gesundheitskarten analog zu gesetzlich Krankenversicherten (das sog. Bremer Modell), die quartalsweise Ausgabe von Behandlungsscheinen und die anlassbezogene Ausgabe von Behandlungsscheinen [31].

Im „Bremer Modell“ erhalten Asylsuchende bereits kurz nach ihrer Ankunft in Deutschland eine Gesundheitskarte [32]. Damit können sie medizinische Leistungen in Anspruch nehmen, wobei der Leistungskanon bis auf wenige Ausnahmen dem der GKV ähnelt [33, 34]. Die Abrechnung erfolgt analog zu gesetzlich krankenversicherten Patient/-innen mit der Krankenkasse, die sich die ausgelegten Kosten dann vom zuständigen Sozialamt erstatten lässt. In diesem Modell sind die logistischen Barrieren im Zugang zu medizinischer Versorgung am niedrigsten [35, 36] und die Verwaltungskosten am geringsten [24].

Die beiden anderen Vorgehensweisen gleichen sich darin, dass Asylsuchende keine Gesundheitskarte erhalten, sondern ihren Status durch vom Sozialamt ausgehändigte Behandlungsscheine nachweisen. Der Behandlungsschein wird den behandelnden Ärzt/-innen vorgelegt und die Behandlung dann beim zuständigen Sozialamt abgerechnet [37]. Medizinische Leistungen, die nach § 4 AsylbLG vom Sozialamt übernommen werden müssen, können ohne vorherige Rücksprache mit dem Sozialamt erbracht und dann abgerechnet werden. Bei Behandlungen, die nach § 6 AsylbLG vom Sozialamt übernommen werden können, muss vor Beginn der Behandlung ein Antrag auf Kostenübernahme gestellt werden, wenn die Behandlungskosten vom Sozialamt getragen werden sollen. Wird die Kostenübernahme nicht vor Behandlungsbeginn beantragt und genehmigt, kann das zuständige Sozialamt die Kostenübernahme verweigern [38].

Im Fall der quartalsweisen Ausgabe der Behandlungsscheine werden diese zu Quartalsbeginn an die Asylsuchenden ausgegeben und können dann im Krankheitsfall sofort verwendet werden [15, 37]. Damit wird sichergestellt, dass Asylsuchende im Krankheitsfall direkt ärztliche Behandlung in Anspruch nehmen können, ohne zuvor erst das Sozialamt aufsuchen zu müssen und ggf. von dessen Öffnungszeiten abhängig zu sein.

Im Fall anlassbezogener Behandlungsscheine hingegen müssen die Behandlungsscheine im konkreten Krankheitsfall bei dem zuständigen Sozialamt beantragt werden und werden erst ausgegeben, nachdem das Sozialamt die Notwendigkeit einer Behandlung geprüft hat [23, 39].

Neben der damit einhergehenden Stigmatisierung der Patient/-innen [23, 39] impliziert dieses Vorgehen, dass in diesen Fällen der Zugang zu medizinischer Versorgung in hohem Maße von der Entscheidung medizinischer Laien abhängig ist. Dies wird von vielen Seiten, u. a. von der Bundesärztekammer, seit vielen Jahren kritisiert [40], da hieraus erhebliche Barrieren entstehen, die den Zugang zu medizinischer Versorgung erschweren und den Behandlungsbeginn verzögern [23, 39], wobei das Ausmaß der Erschwernis vom zufällig zugewiesenen Wohnort abhängt [18].

### Wohnbedingungen

Asylsuchenden wird jedoch nicht nur ihr Wohnort vorgeschrieben, sondern auch die Unterbringung in bestimmten Unterkünften. Während es an einigen Orten Ansätze gibt, Asylsuchende in eigenen Wohnungen unterzubringen, ist die Unterbringung in Sammelunterkünften im Allgemeinen nach wie vor die Norm [41]. Hierbei zeigt sich, dass viele Asylsuchende in Sammelunterkünften mit niedriger baulicher Qualität leben [42] und die Sammelunterkünfte überdurchschnittlich häufig in deprivierten Stadtteilen liegen, in denen der Zugang zu gesellschaftlicher Infrastruktur schlecht und der soziale Problemdruck hoch ist [43].

So müssen die Bewohner/-innen häufig weite Wege zurücklegen, um medizinische Versorgung in Anspruch zu nehmen [23]. Zudem werden soziale Integration, der Erwerb der deutschen Sprache und eine Vielzahl von Freizeitaktivitäten – Faktoren, deren gesundheitsfördernde Bedeutung unstrittig ist – durch die geographische Lage der Sammelunterkünfte häufig erschwert oder verhindert [44].

Qualitative Arbeiten zeigen entsprechend, dass der Mangel an Freizeitaktivitäten und Privatsphäre die Bewohner/-innen von Sammelunterkünften belastet und zu Konflikten führt [45, 46]. Sammelunterkünfte werden von den Bewohner/-innen häufig als „totale Institutionen“ beschrieben, die ihr Leben fremdbestimmen und die Erfahrung von Selbstwirksamkeit unterdrücken [45]. Neben dem sozialen Arrangement der Unterkünfte liegt dies auch an der gebauten Umwelt der Einrichtungen, bei denen es sich zum Teil um frühere Kasernen handelt, die in ihrer Architektur auf die Disziplinierung der Bewohner/-innen ausgerichtet sind [45, 47].

Während der COVID-19-Pandemie haben sich diese Zustände noch weiter zugespitzt. Obwohl das *Kompetenznetz Public Health COVID-19 *in seinen Empfehlungen für das Pandemiemanagement in Sammelunterkünften für Geflüchtete explizit von Kollektivquarantäne abgeraten hat [48], wurde dieses epidemiologisch vermutlich unwirksame Mittel vielfach eingesetzt [49, 50] und war für die Bewohner/-innen der Einrichtungen mit besonderen Belastungen verbunden [46, 51]. Insbesondere der verstärkte Einsatz von Sicherheitspersonal und Polizei evozierte Erinnerungen an die Situation in polizeistaatlichen Herkunfts- oder Transitländern und näherte die Situation in Sammelunterkünften aus Sicht der Bewohner/-innen an die Verhältnisse in Gefängnissen an [46]. Zudem fielen durch die Pandemiemaßnahmen häufig Unterstützungsangebote weg, die vor der Pandemie darauf abzielten, die Belastungen der Unterbringung in Sammelunterkünften abzufedern [51].

### Weitere Determinanten

Neben diesen Einflussfaktoren, die sich aus dem Aufenthaltsstatus von Asylsuchenden ableiten, gibt es weitere Determinanten, die aus dem im deutschen Gesundheitssystem prävalenten Mangel an diversitätssensibler Versorgung resultieren, der andere marginalisierte Patient/-innen ebenfalls betrifft und sich in der Versorgung von Asylsuchenden vor allem in Bezug auf ihre Navigation im Gesundheitssystem, den Umgang mit Sprachbarrieren und die Konfrontation mit rassistischen und kulturalisierenden Stereotypen zeigt.

Innerhalb des Gesundheitssystems werden Abläufe häufig als selbstverständlich vorausgesetzt und daher nicht expliziert [23]. Asylsuchende erhalten zudem zu keinem Zeitpunkt im Asylverfahren strukturierte Informationen über die Mechanismen des deutschen Gesundheitssystems [52], sodass es häufig eine Frage des Zufalls ist, ob Asylsuchende im konkreten Krankheitsfall über die nötigen Informationen verfügen, um z. B. einen Behandlungsschein des Sozialamtes zu bekommen [23], die ihnen rechtlich zustehenden Leistungen einzufordern [53] oder die für ihre Beschwerden richtige Ansprechperson innerhalb des Gesundheitssystems zu finden [52, 53].

Hinzu kommt, dass Kontakte zum Gesundheitssystem häufig durch eine Sprachbarriere geprägt sind, da das deutsche Gesundheitssystem nach wie vor keine Mechanismen zum systematischen und evidenzbasierten Umgang mit sprachdiskordanten Behandlungssituationen etabliert hat [54]. Studien zeigen, dass professionelles Dolmetschen im Gesundheitssystem die Zufriedenheit von Ärzt/-innen und Patient/-innen verbessert [55, 56], ungeplante stationäre Aufnahmen vermindert [57], die Informiertheit der Patient/-innen erhöht [56, 58], die Inanspruchnahme von Vorsorgeuntersuchungen steigert [56], die Anzahl diagnostischer Tests zur Diagnosestellung senkt [55, 57] und insgesamt Kosten einspart [56, 57].

Bisher hat dies jedoch nicht dazu geführt, dass der Einsatz professioneller Dolmetscher/-innen im Alltag selbstverständlich geworden wäre. Zwar gibt es vereinzelt Ansätze, z. B. professionelle Telefon- oder Videodolmetscher in sprachdiskordante Arzt-Patienten-Gespräche einzubinden [59]. Zumeist werden im klinischen Alltag aber nach wie vor Laien (z. B. Angehörige oder Bekannte der Patient/-innen, Krankenhauspersonal oder zufällig Anwesende) eingesetzt [60], obwohl in der Literatur aufgrund der hohen Fehlerquote und der niedrigen Qualität der Übersetzung sowie wegen der damit verbundenen Probleme in Bezug auf die Vertraulichkeit einhellig vom Einsatz von Laien abgeraten wird [58].

Die Etablierung dieser der bestehenden Evidenz zuwiderlaufenden Praxis hat einerseits Abrechnungsgründe, da professionelle Dolmetscher/-innen von der GKV nicht bezahlt werden. Leistungserbringer stehen daher vor der Herausforderung, Dolmetschkosten querfinanzieren zu müssen oder die Kostenübernahme in jedem Einzelfall mit erheblichem administrativen Aufwand bei dem zuständigen Sozialamt bzw. Jobcenter zu beantragen [61].

Andererseits deutet die stillschweigende Akzeptanz einer erwiesenermaßen zu schlechteren Behandlungsergebnissen führenden Praxis – und die Verweigerung der Übernahme von Verantwortung für die sich daraus ergebenden Probleme [30] – auf die Durchdringung klinischen Handelns mit nichtmedizinischen Logiken hin. So betont Fassin [62] die Bedeutung moralischer Hierarchien in der Versorgung von Asylsuchenden. Diese Hierarchien sind daran gebunden, ob den Patient/-innen im Selbstverständnis des medizinischen Personals zugeschrieben wird, medizinische Versorgung zu verdienen, bzw. ob Versorgungsdefizite sich vor dem Hintergrund der ihnen in der moralischen Hierarchie zugewiesenen Position rechtfertigen lassen [63]. In der Praxis kann dies dazu führen, dass Ärztinnen und Ärzte in der Versorgung Asylsuchender (oder anderer marginalisierter Patient/-innen) eher bereit sind, von etablierten Versorgungsstandards abzuweichen [63], wodurch Medizin in den Wirkungskreis ihr zunächst fremder, z. B. politischer Interessen geraten kann [64]. Für eine ausführliche Auseinandersetzung mit dieser Problematik siehe den Beitrag zu strukturellen Determinanten der Gesundheit von Migrant/-innen in diesem Themenheft.

## Gesundheitliche Situation von Asylsuchenden

Die rechtliche Sonderstellung von Asylsuchenden impliziert also Einschränkungen der Lebensgestaltung, aus denen häufig prekäre Lebensbedingungen resultieren. Die Literatur bezeichnet diese – zusammen mit anderen Belastungen, wie z. B. der Konfrontation mit Rassismus und gesellschaftlicher Ausgrenzung, der Unsicherheit des Asylverfahrens und der sich daraus ergebenden unklaren Zukunftsperspektive oder dem mit Migration häufig einhergehenden Statusverlust – als postmigratorische Stressoren [65].

Die postmigratorischen Stressoren wirken mit prämigratorischen Stressoren (wie den oft desolaten Gesundheitssystemen der Herkunftsländer oder dem Leben unter Bedingungen, die durch Armut, politische Verfolgung oder andere Formen von Gewalt geprägt sind) und perimigratorischen Stressoren (wie der Unsicherheit und den Belastungen während der Flucht, Gewalterfahrungen und mangelhafter medizinischer Versorgung) zusammen [66], woraus einige Besonderheiten sowohl in Bezug auf das unter Asylsuchenden zu beobachtende Krankheitsspektrum als auch die Inanspruchnahme medizinischer Leistungen resultieren.

### Krankheitsspektrum

Asylsuchende nehmen das Gesundheitssystem im Wesentlichen mit den gleichen Erkrankungen wie die Allgemeinbevölkerung in Anspruch [6, 67–69]. Dennoch gibt es einige Besonderheiten, wobei vorrangig psychische Erkrankungen zu nennen sind [42, 70].

In großer Übereinstimmung zeigen internationale Studien [71] und Studien aus Deutschland, dass die Prävalenzen vor allem für Depression, Angststörung und posttraumatische Belastungsstörung (PTBS) bei Asylsuchenden deutlich höher sind als in der Allgemeinbevölkerung [72, 73], wobei der Vergleich mit anderen Gruppen von Migrant/-innen zeigt, dass insbesondere die mit dem Asylverfahren verbundenen Unsicherheiten und die regelhaft prekären Lebensbedingungen während des Asylverfahrens (sowie während des Aufenthaltes in Deutschland mit einer Duldung) eine große Rolle für die Häufigkeit psychischer Erkrankungen spielen [74–76]. Hierbei ist zu betonen, dass die Unsicherheit des Asylverfahrens und die sich daraus ergebende unklare Zukunftsperspektive [76, 77] einerseits vorbestehende gesundheitliche Beeinträchtigungen verstärken, gleichzeitig aber auch die Therapie erschweren und damit insgesamt einen deutlich negativen Effekt auf die Prognose psychischer Erkrankungen haben [75, 76, 78–80]. Auch wird davon ausgegangen, dass die erhöhte Prävalenz von psychischen Störungen wie etwa Depression unter Asylsuchenden dadurch maßgeblich mitverursacht wird [81, 82].

So liegt die Prävalenz von Depression, Angststörung und posttraumatischer Belastungsstörung in der deutschen Allgemeinbevölkerung bei 9,8 %, 15,4 %, bzw. 2,3 % [83]. Im Vergleich dazu werden für verschiedene Kohorten von Asylsuchenden Prävalenzen zwischen 28 % und 75 % für Depression [70, 84], 45 % für Angststörungen [70] und bis zu 41 % für PTBS [84, 85] beschrieben. Dabei zeigt sich eine hohe Komorbidität der Erkrankungen [72]. Studien mit Stichproben aus Erstaufnahmeeinrichtungen, die Asylsuchende am Anfang ihres Aufenthaltes in Deutschland einschließen, finden ähnliche [69] oder sogar noch höhere Prävalenzen [86].

In Ermangelung strukturierter Mechanismen zur Identifizierung von psychischen Erkrankungen werden diese jedoch bei vielen Asylsuchenden übersehen [87] und selbst im Fall der diagnostischen Abklärung psychischer Beschwerden erfolgt häufig keine oder keine adäquate therapeutische Versorgung innerhalb des Gesundheitssystems [87], sodass viele Asylsuchende auf Versorgungsangebote außerhalb der Regelversorgung, z. B. durch die psychosozialen Zentren für Migrant/-innen (PSZ), angewiesen sind. Diese Zentren sind einerseits auf die Versorgung von Patient/-innen mit Migrationserfahrung spezialisiert, bieten daher routinemäßig Therapien unter Einsatz von Dolmetscher/-innen an und arbeiten mit interkulturell sensiblen therapeutischen Zugängen [77]. Andererseits haben auch die PSZ lange Wartelisten [88] und stellen potenziell eine Parallelstruktur zur Regelversorgung dar, der durch die Integration spezialisierter Angebote in die Regelversorgung eigentlich vorgebeugt werden sollte.

Neben der erhöhten Prävalenz psychischer Erkrankungen zeigt sich bei Asylsuchenden zudem konsistent eine schlechte subjektive Gesundheit [42, 89].

Darüber hinaus ist zu erwähnen, dass insbesondere die häufig defizitäre medizinische Versorgung in den Herkunftsländern vieler Asylsuchender zu einer mangelhaften Abdeckung mit präventivmedizinischen Angeboten wie Impfungen führt [72], sodass Asylsuchende einer erhöhten Gefahr für impfpräventable und andere Infektionskrankheiten ausgesetzt sind [90]. Dieses Risiko wird noch einmal erhöht durch die Unterbringung in Sammelunterkünften. Dies hat in der Vergangenheit in Sammelunterkünften für Asylsuchende immer wieder zu Ausbrüchen von Masern [91–94], Windpocken [95, 96], Scabies [97] und Noroviren [98] geführt und im Rahmen der COVID-19-Pandemie Asylsuchende in Sammelunterkünften einem im Vergleich zur Allgemeinbevölkerung deutlich erhöhten Ansteckungsrisiko ausgesetzt [48, 50].

### Inanspruchnahme medizinischer Leistungen

Aus den eingangs geschilderten rechtlich bedingten Zugangsbarrieren und den Problemen bei der Navigation im Gesundheitssystem ergeben sich einige Besonderheiten der Inanspruchnahme des Gesundheitssystems durch Asylsuchende.

In der Literatur vordergründig diskutiert werden dabei die Konsequenzen der „Krankenscheinbürokratie“ [39]: Die durch die Behandlungsscheine entstehenden hohen Hürden im Zugang zu medizinischer Versorgung gehen einher mit einer geringen Inanspruchnahme ambulanter und präventiver Leistungen [35, 70, 99] sowie fachärztlicher Versorgung [37], während notfallmäßige Vorstellungen, Inanspruchnahme des Rettungsdienstes und stationäre Aufenthalte häufiger sind als bei gesetzlich Krankenversicherten [42, 68, 89]. Diese Unterschiede in der Inanspruchnahme medizinischer Leistungen verschwinden, wenn Asylsuchende eine Gesundheitskarte bekommen [35, 37].

Die Behandlungsscheinbürokratie und ihre Effekte auf das Inanspruchnahmeverhalten führen zu höheren Kosten, sowohl für das Gesundheits- und Sozialsystem [10, 100] als für auch die betroffenen Individuen [23, 75]. Gründe dafür sind vermutlich, dass es bei eingeschränktem Zugang zu medizinischer Versorgung durch die Chronifizierung von Beschwerden und die Verschiebung des Behandlungsbeginns in spätere Erkrankungsstadien bei vielen Krankheiten zu einer Verschlechterung der Prognose kommt bzw. aufwändigere Therapien nötig sind, was dazu führt, dass sich Behandlungen tendenziell aus dem (relativ kostengünstigen) ambulanten Sektor in den stationären Sektor verschieben [10, 18, 100].

## Fazit

Um die Gesundheit von Asylsuchenden zu verbessern, sollten die sozialen, rechtlichen und politischen Determinanten ihrer Gesundheit zum Ausgangspunkt genommen und vordringlich auf diesen Ebenen Optimierungen angestrebt werden [101]. Im Zentrum sollten dabei die Beendigung der sozialrechtlichen Sonderstellung von Asylsuchenden und die Förderung ihrer vollständigen Integration in das deutsche Sozialsystem stehen [102]. Die Einführung der elektronischen Gesundheitskarte für Geflüchtete auf kommunaler oder Länderebene kann ein Zwischenschritt auf diesem Weg sein [103].

Neben den rechtlichen und administrativen Einschränkungen im Zugang zu medizinischer Versorgung müssen die mit erhöhten gesundheitlichen Risiken verbundenen Lebens- und Wohnbedingungen in Sammelunterkünften adressiert werden. Vor dem Hintergrund des gesicherten Wissens zu den krankmachenden Effekten dieser Art von Unterbringung sollte angestrebt werden, Asylsuchende dezentral unterzubringen und lagerähnliche Sammelunterkünfte zu vermeiden [44, 104]. Die aktuelle politische Diskussion, die einen Ausbau von Ankerzentren^4^ fordert, geht hierbei aus sozialmedizinischer Sicht eindeutig in die falsche Richtung.

Innerhalb des Gesundheitssystems sollten systematisch Mechanismen zum evidenzbasierten Umgang mit sprachdiskordanten Patientenkontakten etabliert werden [105]. Dies kann einerseits durch lokale Lösungen vorangetrieben werden, setzt aber für eine nachhaltige und flächendeckende Verwirklichung hochwertiger Versorgung eine Kostenübernahme von Dolmetschleistungen voraus, wie sie im Koalitionsvertrag 2021–2025 der Ampel-Regierung angekündigt ist [106]. Auf Ebene des medizinischen Personals sollten Aus- und Weiterbildungsmaßnahmen etabliert werden, die die strukturelle Kompetenz der Ärzteschaft und anderer medizinischer Berufe verbessern und die Entstehung von moralischen Hierarchien verhindern, die medizinethischen Prinzipien zuwiderlaufen [107].

## Footnotes

1 Diese Übersichtsarbeit enthält überarbeitete Ausschnitte aus der Habilitationsschrift des Autors [4].

2 In dieser Arbeit beschreibt der Begriff *Asylsuchende* Menschen, die einen Asylantrag gestellt haben und deren Asylverfahren noch läuft oder deren Asylantrag abgelehnt wurde, deren Abschiebung aber ausgesetzt wurde. Damit umfasst der Begriff Menschen mit *Aufenthaltsgestattung* und *Duldung*, da beide Aufenthaltsstatus zum Bezug von Leistungen nach Asylbewerberleistungsgesetz berechtigen. In der Folge wird der Begriff Asylsuchende in Bezug auf Erwachsene benutzt. Unbegleitete minderjährige Flüchtlinge werden hier nicht thematisiert. Für Ausführungen dazu siehe [5].

3 Beide Zielstellungen erlebten im Zuge der Diskussionen im Kontext der sog. Flüchtlingskrise 2015 und 2016 eine Renaissance. Gleichzeitig konnten wissenschaftliche Arbeiten aber zeigen, dass die sog. Pull-Faktoren in der Modulation internationaler Migration nur eine sehr untergeordnete Rolle spielen [9], während die Einschränkung von Leistungen keine Kosten spart, sondern Mehrkosten verursacht [10].

4 Ankerzentren sind zentrale Aufnahmestellen für Asylsuchende in Deutschland, in denen Asylsuchende während des gesamten Asylprozesses untergebracht sind und die sie erst verlassen, wenn ihr Asylantrag bewilligt wurde oder sie abgeschoben werden. AnkER steht hierbei für „Ankunft, Entscheidung und Rückführung“ [44].
